# Activity modifies adult brain maturity

**DOI:** 10.18632/oncotarget.18560

**Published:** 2017-06-18

**Authors:** Katsunori Kobayashi

**Affiliations:** Department of Pharmacology, Graduate School of Medicine, Nippon Medical School, Bunkyo-ku, Tokyo, Japan

**Keywords:** hippocampus, dentate gyrus, development, antidepressant, electroconvulsive therapy

Neuronal activity plays a critical role in regulating development of the central nervous system. In general, neuronal excitation is known to promote maturation of immature developing neurons [1, 2]. Imoto et al. (2017) recently reported a distinct activity-dependent regulation of neuronal maturation in the adult mouse brain [3]. The authors have demonstrated that electroconvulsive stimulation (ECS) changes the phenotype of mature granule neurons in the hippocampal dentate gyrus to immature-like one. The phenotypic changes caused by ECS included a strong decrease in the expression level of mature neuronal markers, suppression of stimulus-induced expression of immediate early genes, and emergence of immature-like electrophysiological properties such as higher excitability. Single ECS rapidly induced some of these “demature” phenotypes via activation of glutamate NMDA receptors, and repeated stimulation established the robust and long-lasting phenotypic changes. After the period of repeated ECS, augmentation of GABA_A_ receptor-mediated inhibition by diazepam facilitated recovery to the initial matured state, suggesting that stimulus-independent endogenous neuronal activity supports the demature phenotype. The authors concluded that brief neuronal activation by ECS induces dematuration of the fully matured granule neurons and that the enhanced excitability maintains their demature state probably in a cell-autonomous manner.

Electroconvulsive stimulation is an animal model of electroconvulsive therapy for psychiatric disorders including depression. Chronic administration of the antidepressant fluoxetine has also been shown to induce dematuration of the dentate granule cells in adult mice [4]. Both ECS and fluoxetine can strongly enhance monoaminergic modulation at the granule cell output synapse in adult mice as well [4-6]. These marked similarities between the effects of ECS and fluoxetine suggests that altered functioning of the mature dentate granule cells is commonly involved in the cellular mechanisms of physical and chemical antidepressant treatments. Electroconvulsive therapy has fast-acting antidepressant effects and is effective in medication-resistant patients. Similarly, ECS can more rapidly and reliably induce dematuration of the granule cells as compared with fluoxetine [3, 4]. In addition, excessive dematuration is associated with aberrant behavior resembling antidepressant-induced mania [7]. Therefore, the granule cell dematuration well models both therapeutic effects and adverse reaction of antidepressant treatments.

The activity-dependent regulation of the neuronal maturation status demonstrated by Imoto et al. (2017) is in contrast to the known role of excitation, including intense activity caused by seizures, in facilitating structural and functional maturation of the dentate granule cells [1, 2]. This apparent discrepancy is most likely due to the difference in the maturational stage of neurons. While the facilitatory effects of excitation are typically observed in early postmitotic neurons, dematuration by ECS is induced in fully matured neurons. Therefore, the effect of excitation on the maturation status of the granule cells appears to change in direction during development. Although the exact timing of such switching is unknown, the bidirectional effects of excitation on the granule cell maturation suggest that both excessive and insufficient excitation can affect normal maturational processes of these neurons depending on their developmental stage and could potentially cause a failure in their maturation in the adult brain. The impaired granule cell maturation has been suggested to be an endophenotype of psychiatric disorders such as schizophrenia [8]. Altered cellular functioning due to failure in neuronal maturation would cause dysfunction of neuronal circuits in the adult brain and may serve as the pathophysiological basis of psychiatric disorders. The demature state caused by repeated ECS was supported by neuronal activity and augmented synaptic inhibition promoted rematuration to the initial matured state [3], which suggests that the altered state of maturation of the brain neurons could be controlled by changing an excitation/inhibition balance in adults. Increased inhibition may improve aberrant neuronal maturation caused by heightened excitability, as in ECS-treated demature granule cells. However, it may exacerbate perturbed neuronal maturation caused by insufficient excitation. In addition to neuronal activity, inflammation and monoaminergic modulation have been implicated in the modifications of the maturation status of adult hippocampal neurons [4, 8]. To reveal heterogeneous pathophysiological mechanisms of psychiatric disorders and to devise effective treatments for them, it would be of critical importance to understand the cell stage-dependent regulation of neuronal maturation by activity and other factors.

**Figure 1:**
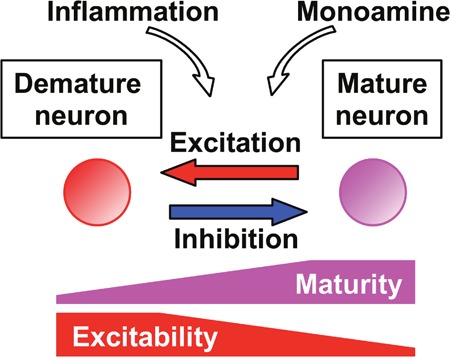
Regulation of neuronal maturity level by activity and other factors
